# A review of Norwegian *Gymnometriocnemus* (Diptera, Chironomidae) including the description of two new species and a new name for *Gymnometriocnemus volitans* (Goetghebuer) sensu Brundin

**DOI:** 10.3897/zookeys.508.9874

**Published:** 2015-06-17

**Authors:** Elisabeth Stur, Torbjørn Ekrem

**Affiliations:** 1 Norwegian University of Science and Technology, NTNU University Museum, Department of Natural History, NO-7491 Trondheim, Norway

**Keywords:** Chironomidae, Orthocladiinae, DNA barcodes, new species, taxonomy, non-biting midges

## Abstract

Examination of the syntypes of *Metriocnemus volitans* Goetghebuer, 1940 revealed that these specimens belong to the genus *Chaetocladius* and are not con-specific with *Gymnometriocnemus volitans* (Goetghebuer, 1940) sensu Brundin (1956) and Sæther (1983). A literature search showed that *Gymnometriocnemus kamimegavirgus* Sasa & Hirabayashi, 1993 fits well with the species figured and diagnosed by Brundin (1956) as well as with specimens of this species from Norway. We present arguments for *Chaetocladius volitans* (Goetghebuer) comb. n. and for the use of *Gymnometriocnemus kamimegavirgus* for *Gymnometriocnemus volitans* sensu Brundin. In addition, we provide DNA barcode data that indicate the presence of at least seven *Gymnometriocnemus* species in Norway of which six are collected as male adults. Two of these, Gymnometriocnemus (Gymnometriocnemus) pallidus **sp. n.** and Gymnometriocnemus (Raphidocladius) autumnalis **sp. n.** are regarded as new to science and diagnosed based on adult male morphology and DNA barcodes. The species Gymnometriocnemus (Gymnometriocnemus) marionensis Sæther, 1969 is re-established and a key to all Holarctic species is provided.

## Introduction

The orthoclad genus *Gymnometriocnemus* was suggested by Goetghebuer (1932), but without designation of a type species. Edwards (1932) designated *Gymnometriocnemus subnudus* (Edwards, 1929) as type species and made the name available according to the International Code of Zoological Nomenclature. Edwards is thus credited authorship of the genus (Spies and Sæther 2004). The genus was revised by Sæther (1983) who distinguished two subgenera based on adult male and pupal morphology. There currently are 15 *Gymnometriocnemus* species recognized (Ashe and O’Connor 2012). Larvae of most species have previously been regarded as terrestrial (Andersen et al. 2013), but there is evidence for at least semi-aquatic lifestyle in larvae of subgenus *Raphidocladius* from Norway (own data).

*Metriocnemus volitans* was described by Goetghebuer (1940) based on material collected in Abisko, Sweden by Thienemann in 1939. The species was placed in genus *Gymnometriocnemus* by Brundin (1956), probably without consulting the type material. Since then, chironomid workers have used Brundin’s interpretation of this species and even Sæther (1983) in his revision of Holarctic *Gymnometriocnemus* relied on the characters presented by Brundin (1956). He stated explicitly, however, that he had not examined Goetghebuer’s types.

Sæther (1983) erected the subgenus *Raphidocladius* for *Gymnometriocnemus* species possessing an extremely long virga with needle-like sclerotization in the adult males. The species *Gymnometriocnemus brumalis* (Edwards, 1929) and *Gymnometriocnemus acigus* Sæther, 1983 were listed as members of the group, while *Gymnometriocnemus volitans* was considered a possible member since immatures were unknown and virga had not been examined (Sæther 1983). Two species in this subgenus were later described from Japan, but both *Gymnometriocnemus kamimegavirgus* Sasa & Okazawa, 1994 and *Gymnometriocnemus tairaprimus* Sasa & Okazawa, 1994 were diagnosed without considering *Gymnometriocnemus volitans* sensu Brundin (1956) and Sæther (1983) (Sasa and Hirabayashi 1993; Sasa and Okasawa 1994). Cranston and Oliver (1988) argued for a synonymy of the type species for *Raphidocladius*, Gymnometriocnemus (Raphidocladius) acigus, with Gymnometriocnemus (Raphidocladius) brumalis claiming that the characters used by Sæther (1983) to separate these species were not reliable and interspecifically variable even within the same population. This leaves four valid species in subgenus *Raphidocladius* in the World Catalogue of Chironomidae: Gymnometriocnemus (Raphidocladius) brumalis, Gymnometriocnemus (Raphidocladius) kamimegavirgus, Gymnometriocnemus (Raphidocladius) tairaprimus and Gymnometriocnemus (Raphidocladius) volitans (Ashe and O’Connor 2012).

Subgenus *Gymnometriocnemus* is characterised by a short virga and a weakly developed crista dorsalis in the adult male hypopygium (Sæther 1983). Cranston and Oliver (1988) synonymized *Gymnometriocnemus marionensis* Sæther, 1983 with *Gymnometriocnemus subnudus*, leaving 11 valid species in subgenus *Gymnometriocnemus* the World Catalogue of Chironomidae: Gymnometriocnemus (Gymnometriocnemus) ancudensis (Edwards, 1931), Gymnometriocnemus (Gymnometriocnemus) benoiti (Freeman, 1956), Gymnometriocnemus (Gymnometriocnemus) brevitarsis (Edwards, 1929), Gymnometriocnemus (Gymnometriocnemus) johanasecundus Sasa & Okazawa, 1994, Gymnometriocnemus (Gymnometriocnemus) lobifer (Freeman, 1956), Gymnometriocnemus (Gymnometriocnemus) longicostalis (Edwards, 1931), Gymnometriocnemus (Gymnometriocnemus) subnudus, Gymnometriocnemus (Gymnometriocnemus) terrestris Krüger, Thienemann & Goetghebuer, 1941, Gymnometriocnemus (Gymnometriocnemus) mahensis (Kieffer, 1911) Gymnometriocnemus (Gymnometriocnemus) nitidulus (Skuse, 1889) and Gymnometriocnemus (Gymnometriocnemus) wilsoni Freeman, 1961 (Ashe and O’Connor 2012).

DNA barcoding using partial cytochrome c oxidase subunit 1 sequences (COI) (Hebert et al. 2003) has been shown to perform well for species identification of many Chironomidae groups (Brodin et al. 2012; Ekrem et al. 2010; Ekrem et al. 2007; Stur and Ekrem 2011). We have therefore had a strong focus on developing a barcode library of chironomids for use in taxonomy, life stage association and future environmental monitoring in Norway. In connection with biosurveillance projects in central, eastern and northern Norway, we have barcoded adult specimens of *Gymnometriocnemus* species from various habitats.

The motivation for this study was to clarify the identity of *Gymnometriocnemus volitans* (Goetghebuer), describe hitherto unknown species of *Gymnometriocnemus* and to present the DNA barcodes of Norwegian *Gymnometriocnemus* as a resource for future studies of this genus.

## Material and methods

**Figure 1.**
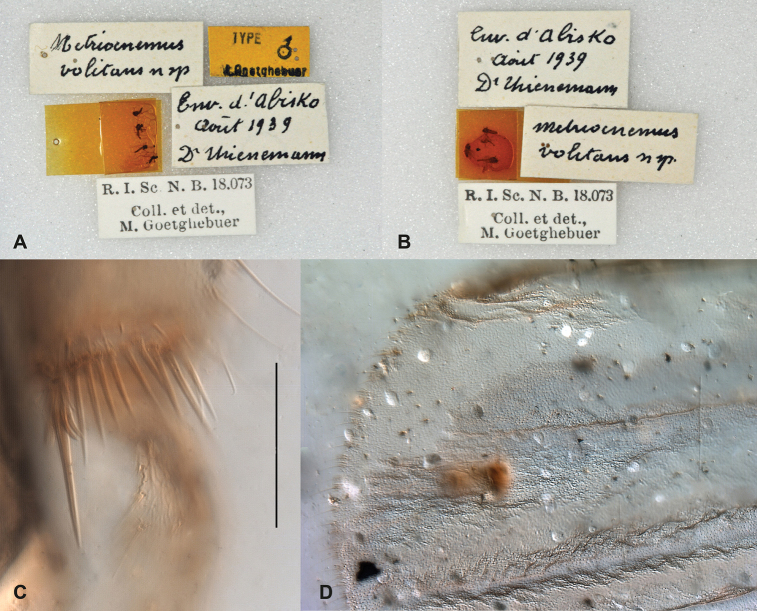
Syntype specimens of *Metriocnemus volitans*. **A, B** specimens and labels on pins **C** hind tibial comb and spur (scale bar = 50 µm) **D** wing tip.

We examined eight syntypes of *Metriocnemus volitans* Goetghebuer from the Royal Belgian Institute of Natural Sciences (RBINS), five male and three female adults mounted between cellophane strips on two separate pins. Both pins bear the label “Env. d. Abisko, Aout 1939, Dr. Thienemann” and “*Metriocnemus volitans* n sp” (Figs 1A-B). Thienemann (1941) uses Goetghebuer’s name and state that only adults were collected in a groundwater spring area at the beginning of the Njulja road 29.viii.1939.

We also examined the male holotype and a male paratype of Gymnometriocnemus (Raphidocladius) acigus Sæther, 1983 (University Museum of Bergen, Norway (ZMBN)) and two male syntypes and a female syntype of Gymnometriocnemus (Gymnometriocnemus) terrestris (RBINS).

Additional material of *Gymnometriocnemus* was collected using a variety of methods in different biosurveillance projects: Malaise traps, sweep netting and fogging of oak canopies (Supplementary file 1). This material is deposited in the NTNU University Museum insect collection (NTNU-VM). One to three legs were dissected off the specimens and submitted to the Canadian Centre for DNA Barcoding. Metadata, photos, sequences and trace-files are available in the Barcode of Life Data Systems (BOLD, www.boldsystems.org) through the dataset DS-GYMNO with doi: 10.5883/DS-GYMNO. GenBank accessions are given in Supplementary file 1.

DNA extracts and partial COI gene sequences were generated using standard primers and bi-directional Sanger sequencing with BigDye 3.1 termination at the Canadian Centre for DNA Barcoding in Guelph. Protocols and original trace-files are available through the dataset DS-GYMNO in BOLD. Alignments were done on amino acid sequences and was trivial as indels were absent; only sequences > 300bp were used in the final alignment. The taxon ID-tree was generated using neighbour joining analysis and 1000 bootstrap replicates on Kimura 2-parameter (K2P) genetic distances in MEGA 6 (Tamura et al. 2013).

Morphological terminology and abbreviations follow Sæther (1980). Antennal and fore leg ratios of Norwegian *Gymnometriocnemus* are given in Table 1. Measurements are given as ranges followed by the mean. Anal point lengths were measured from posterior margin of anal tergite to tip of anal point.

**Table 1.** Antennal ratios (AR) and fore leg ratios (LR_1_) of male *Gymnometriocnemus* from Norway.

Species	AR	LR_1_
Gymnometriocnemus (Raphidocladius) kamimegavirgus	0.88–1.14, 1.04 [n=5]	0.51–0.52, 0.51 [n=3]
Gymnometriocnemus (Raphidocladius) brumalis	1.03–1.31, 1.19 [n=5]	0.50–0.53, 0.51 [n=5]
Gymnometriocnemus (Raphidocladius) autumnalis	0.87–1.00, 0.94 [n=4]	0.56–0.58, 0.57 [n=4]
Gymnometriocnemus (Gymnometriocnemus) subnudus	1.00–1.08, 1.06 [n=3]	0.58–0.63, 0.61 [n=3]
Gymnometriocnemus (Gymnometriocnemus) pallidus	1.05–1.10, 1.07 [n=5]	0.62–0.69, 0.65 [n=5]
Gymnometriocnemus (Gymnometriocnemus) marionensis	1.00–1.08, 1.05 [n=4]	0.60–0.68, 0.65 [n=4]

## Results and discussion

### Chaetocladius volitans

Taxon classificationAnimaliaDipteraChironomidae

(Goetghebuer, 1940) comb. n.

Metriocnemus volitans Goetghebuer, 1940: 59.
Metriocnemus volitans Goetghebuer in Thienemann (1941: 150, 172).

#### Remarks.

**Figure 2.**
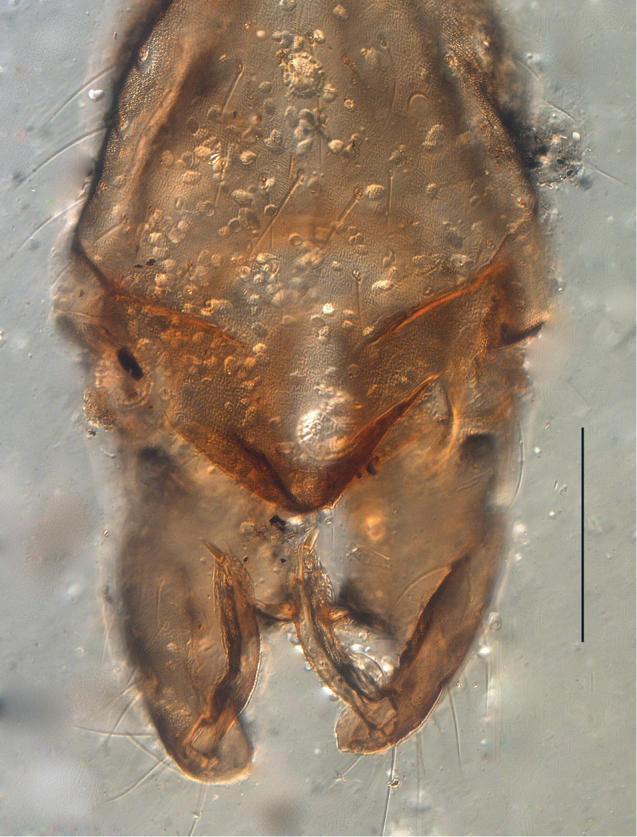
Syntype of *Metriocnemus volitans*, hypopygium (scale bar = 100 µm).

Several of the type specimens are quite damaged and many characters are difficult or impossible to observe (Figs 1–2). However, it is obvious that they do not belong to *Gymnometriocnemus*, and the better preserved male specimens show characters that fits the diagnosis of *Chaetocladius*: wings with coarse punctuation and without macrotrichia on the membrane (Fig. 1D), divergent lateral spinules on the tibial spurs (Fig. 1C) and a fore leg ratio of 0.75. We are confident that this species should be placed in *Chaetocladius*, but due to the state of the specimens and the unrevised nature of the genus, we have not attempted to compare *Chaetocladius volitans* with other species in *Chaetocladius*.

### Gymnometriocnemus brevitarsis

Taxon classificationAnimaliaDipteraChironomidae

Edwards, 1932

#### Remarks.

**Figure 3.**
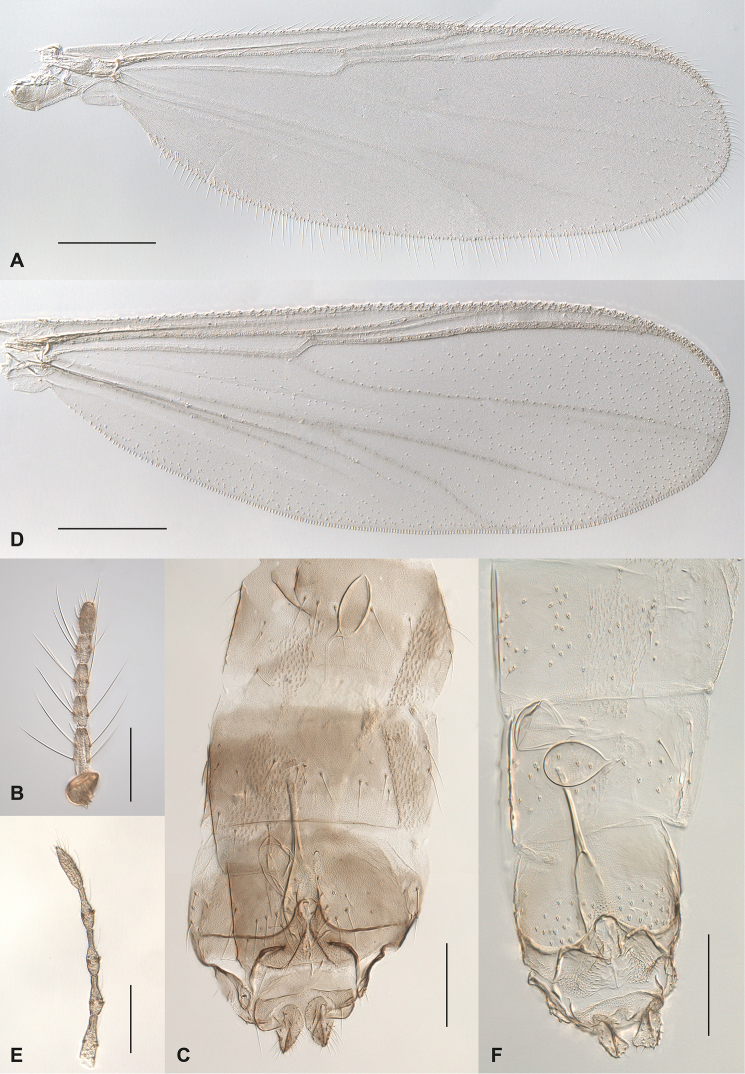
Female *Gymnometriocnemus*. **A–C** *Gymnometriocnemus brevitarsis* (CH-OSF33): **A** Wing **B** Antenna **C** genitalia **D–F** Gymnometriocnemus (Gymnometriocnemus) pallidus sp. n. (CH-eik47): **D** Wing **E** Antenna **F** genitalia. Specimen codes in parenthesis correspond to codes in BOLD and in figure 6. Scale bar: 200 µm (**A, D**); 100 µm (**B, C, E, F**).

We have only seen two females from eastern and central Norway. The two specimens fit Edwards’ description for *Gymnometriocnemus brevitarsis* and represent the first records of this species in Norway. The wing and antenna are photographed (Fig. 3A, B) and leg ratios of fore- mid- and hind legs are measured to be LR_1_ 0.41-0.42 [n=2], LR_2_ 0.38-0.39 [n=2], LR_3_ 0.50-0.53 [n=2] respectively. Only females are known and partial COI gene sequences do not reveal close relationships to any other species in *Gymnometriocnemus*. Subgeneric placement of *Gymnometriocnemus brevitarsis* is therefore not possible.

### Gymnometriocnemus (Gymnometriocnemus) marionensis

Taxon classificationAnimaliaDipteraChironomidae

Sæther, 1969

#### Remarks.

**Figure 4.**
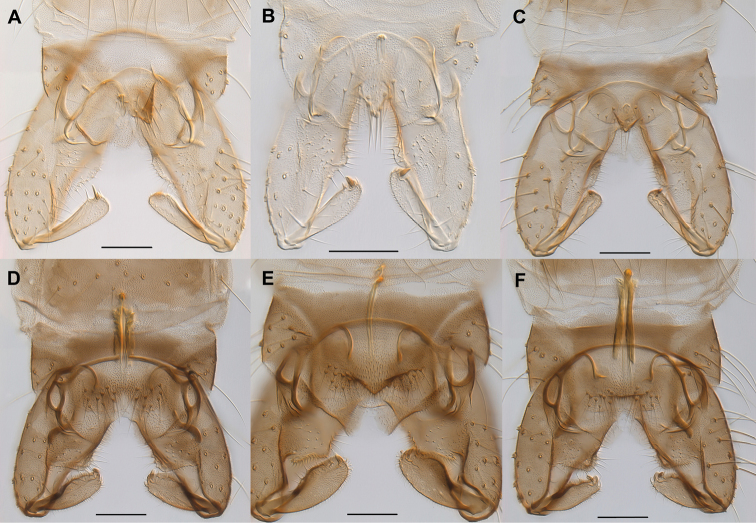
Hypopygia of Norwegian *Gymnometriocnemus*. **A** Gymnometriocnemus (Gymnometriocnemus) marionensis (Finnmark06) **B** Gymnometriocnemus (Gymnometriocnemus) pallidus sp. n. (CH-eik131) **C** Gymnometriocnemus (Gymnometriocnemus) subnudus (ATNA398) **D** Gymnometriocnemus (Raphidocladius) autumnalis sp. n. (Finnmark201) **E** Gymnometriocnemus (Raphidocladius) brumalis (Finnmark75) **F** Gymnometriocnemus (Raphidocladius) kamimegavirgus (Finnmark76). Scale bar = 50 µm. Specimen codes in parenthesis correspond to codes in BOLD and in figure 6.

**Figure 5.**
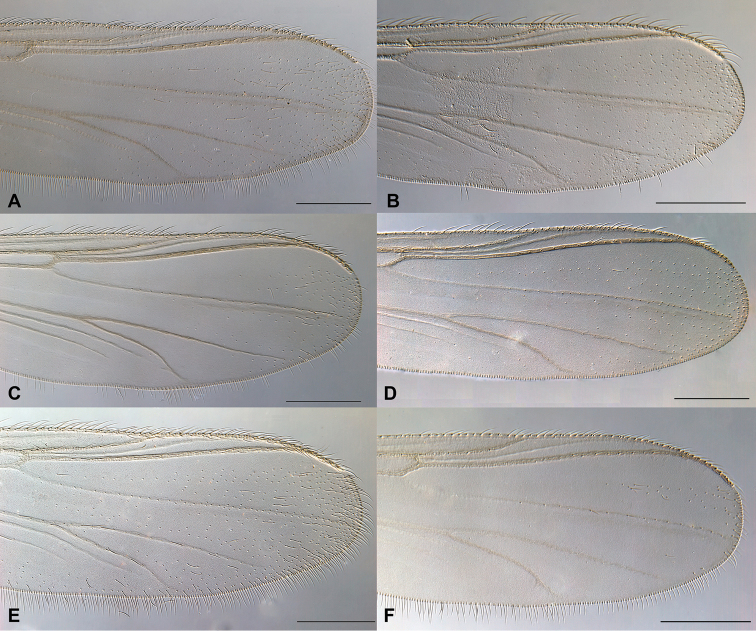
Distal part of wing for Norwegian *Gymnometriocnemus*. **A** Gymnometriocnemus (Gymnometriocnemus) marionensis (Finnmark06) **B** Gymnometriocnemus (Gymnometriocnemus) pallidus sp. n. (CH-eik131) **C** Gymnometriocnemus (Gymnometriocnemus) subnudus (ATNA98) **D** Gymnometriocnemus (Raphidocladius) autumnalis sp. n. (Finnmark201) **E** Gymnometriocnemus (Raphidocladius) brumalis (CH-OSF70) **F** Gymnometriocnemus (Raphidocladius) kamimegavirgus (ATNA269). Scale bar = 200 µm. Specimen codes in parenthesis correspond to codes in BOLD and in figure 6.

Only one specimen of this species from Norway has been available to us. It fits Sæther’s description of Gymnometriocnemus (Gymnometriocnemus) marionensis in having very slightly larger megasetae on the gonostyli than specimens of Gymnometriocnemus (Gymnometriocnemus) subnudus (Figs 4A, C), but this character is not trustworthy as it is dependent on the orientation of the gonostyli in the slide-mount. The larger anal point will separate Gymnometriocnemus (Gymnometriocnemus) marionensis (c. 38 µm long) from *Gymnometriocnemus subnudus* (c. 17 µm long). Cranston and Oliver (1988) synonymized Gymnometriocnemus (Gymnometriocnemus) marionensis with Gymnometriocnemus (Gymnometriocnemus) subnudus doubting the diagnostic value of the size of the megaseta and position of the end of R_2+3_ (Fig. 5A). We have not examined the types of Gymnometriocnemus (Gymnometriocnemus) marionensis, but specimens from Michigan and North Carolina (ZMBN) identified by Ole Sæther as belonging to this species. This record of the species is the first from Norway and Europe.

### Gymnometriocnemus (Gymnometriocnemus) pallidus sp. n.

Taxon classificationAnimaliaDipteraChironomidae

http://zoobank.org/8C19C165-6923-4A17-9C9D-380DD9413E41

#### Type material.

Holotype: Male adult (NTNU-VM slide 143840), Norway, Hordaland, Kvam, Berge, oak canopy fogging, tree #3, 60.30921°N; 6.16453°E, 23.vi.2011, leg. Karl H. Thunes [BOLD ID: CH-eik131]. 5 Paratypes: 2 male adults as holotype except tree #1 60.314°N; 6.167°E, 21.vi.2011; 1 female adult as holotype except tree #18, 59.201°N; 9.920°E, 5.vii.2012; 2 male adults, Norway, Telemark, Porsgrunn, Brevik, Frierflauene, 59.0579°N; 9.66485°E, Malaise trap, 30.vi–27.vii.2010, leg. Geir Søli.

#### Etymology.

The species is named “pallidus”, Latin adjective meaning pale, referring to the conspicuous pale body colour compared to other Holarctic *Gymnometriocnemus*.

#### Diagnosis.

Gymnometriocnemus (Gymnometriocnemus) pallidus can be separated from other *Gymnometriocnemus* species by the following combination of characters in the adults: body pale yellow-green; male with short inconspicuous virga, gonostylus with convex outer margin and weakly developed crista dorsalis. Female with setae on most of wing surface, including numerous in cell m; antenna with apical flagellomere pointed and longer than flagellomere 4; genitalia with long rami, about the same length as notum.

#### Description.

Male adult (n = 5 unless otherwise stated). Wing length 1.21–1.30, 1.28 mm. Colouration pale yellow-green body, legs and antennae; slightly darker bands on scutum; postnotum, dorsal side of head, ventral part of preepisternum pale brown; eyes dark brown.

Head. Antennal ratio in Table 1. Temporal setae 9-10; palp lengths (in µm): 25/30/90-100/80-90 (4)/110-115 (4).

Thorax. Antepronotum with 2-6, 3 setae. Dorsocentrals 10-17, 13; acrostichals 7-12, 10, minute and difficult to discern; prealars 3-4; scutellars 6-7.

Wing (Fig. 5B). Costa moderately extended, not reaching half way to apex of M_1+2_; R_2+3_ approaching costa at 1/3 distance between R_1_ and R_4+5_. Macrotrichia present on membrane in apical half of wing, 0-2 setae in cell m. Veins Sc, R_2+3_, M and pseudovein without setae.

Legs. Fore tibia with one spur, 35 µm long; mid tibia with two spurs ca. 20-25 µm long; hind tibia with well-developed comb and 2 spurs, ca. 20 and 35 µm long. Fore leg ratios in Table 1.

Hypopygium (Fig. 4B). Ninth tergite with 10-15, 13 setae, median setae stronger and situated on an obvious anal point. Virga 15–20 µm long consisting of two spines. Inferior volsella well-developed lobe; gonostylus with slightly convex outer margin.

Female adult (n = 1). Wing length 1.23 mm. Colouration as male.

Head. Antenna (Fig. 3E) with five flagellomeres, lengths (in µm): 75/60/70/60/70. Temporal setae 9; palp lengths (in µm): 25/30/95/-/-.

Thorax. Antepronotum with 6 setae. Dorsocentrals 18; acrostichals 11; prealars 3; scutellars 6.

Wing (Fig. 3D). Costa well extended, reaching slightly past half way to apex of M_1+2_; R_2+3_ approaching costa at 1/3 distance between R_1_ and R_4+5_. Macrotrichia present on membrane in whole wing. Veins M, Sc and R_2+3_ without setae.

Legs. Fore tibia with one spur, 20 µm long; mid tibia lost; hind tibia with well-developed comb and 2 spines, ca. 35–40 µm long. Tarsus of fore leg lost (LR_1_ not measurable).

Genitalia (Fig. 3F). Gonocoxite IX with 7 setae. Ninth tergite undivided, semi-circular with 12 setae; cercus 50 µm long; seminal capsules about 70 µm long and 45 µm wide, seminal tubules about 325 µm long. Notum as long as rami, 87 µm. Inner lobe of gonapophysis VIII broadly rounded with numerous long medially directed microtrichiae.

Immature stages unknown

#### Remarks.

The species is morphologically similar to Gymnometriocnemus (Gymnometriocnemus) subnudus and Gymnometriocnemus (Gymnometriocnemus) johanasecundus, but paler (see whole specimen figures in BOLD dataset DS-GYMNO). Males and females are almost completely yellow-green with pale brown posterior side of head and postnotum; pale brown scutal bands and ventral side of preepisternum. Gymnometriocnemus (Gymnometriocnemus) pallidus is also similar to these species in having a short, triangular anal point and a small virga, but the hypopygium of Gymnometriocnemus (Gymnometriocnemus) pallidus has a more prominent inferior volsella than Gymnometriocnemus (Gymnometriocnemus) johanasecundus and considerably stronger anal tergite setae than Gymnometriocnemus (Gymnometriocnemus) subnudus (Figs 4B, C). Comparison with DNA barcode data in BOLD indicates that the species also has records from Germany and France.

### Gymnometriocnemus (Gymnometriocnemus) subnudus

Taxon classificationAnimaliaDipteraChironomidae

(Edwards, 1929)

#### Remarks.

**Figure 6.**
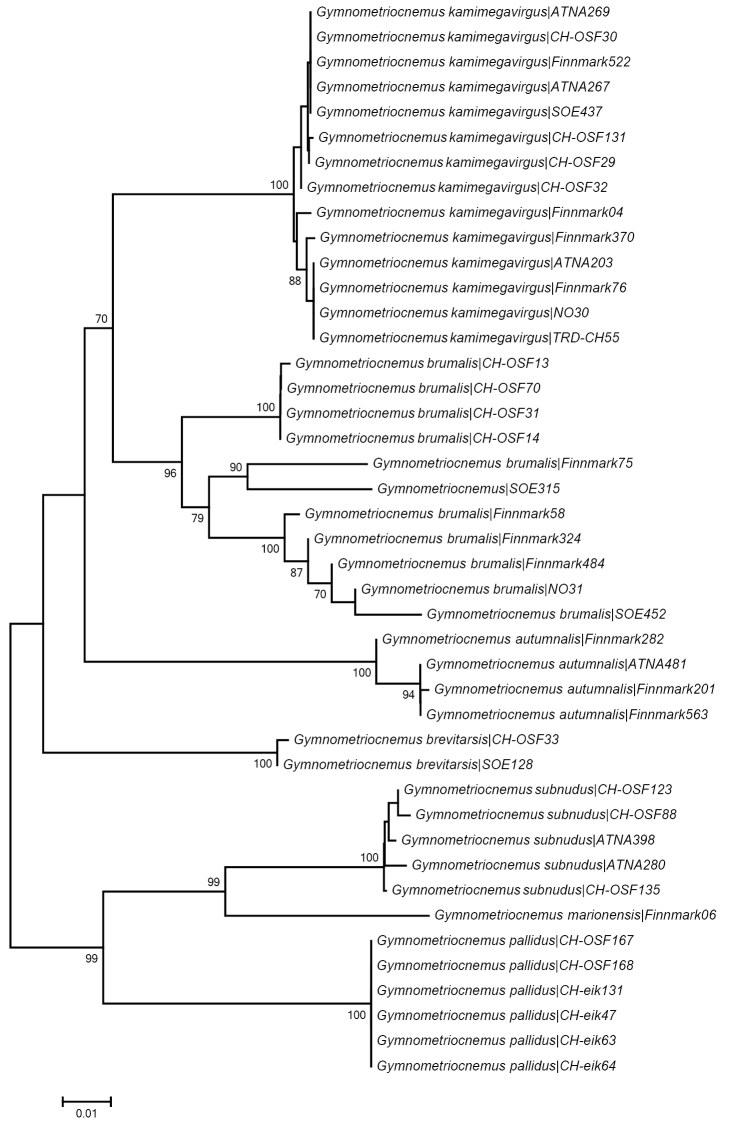
Taxon ID-tree from Neighbour Joining analysis on K2P-distances using 1000 bootstrap replicates. Bootstrap values >70 are given on branches.

We have seen specimens from eastern and central Norway that fit well with the original and later descriptions of the species, except for having a lower AR (1.0-1.1) compared to what Edwards (1929) reported. The specimens group in a well-defined barcode cluster (Fig. 6) separated from Gymnometriocnemus (Gymnometriocnemus) marionensis and Gymnometriocnemus (Gymnometriocnemus) pallidus sp. n. and is fairly widely distributed throughout Europe.

### Gymnometriocnemus (Raphidocladius) autumnalis sp. n.

Taxon classificationAnimaliaDipteraChironomidae

http://zoobank.org/CAB0F99B-1A0A-4078-A68D-D8DB31D0DEB1

Figs 4D , 5D

#### Type material.

Holotype: Male adult (NTNU-VM slide no. 136299), Norway, Finnmark, Porsanger, small pond near Gaggavann, 69.8306°N; 25.1856°E, 107 m a.s.l., 03.ix.2010, leg. Alyssa Anderson [BOLD ID: Finnmark201]. 3 Paratypes, male adults: 1 Norway, Finnmark, Vardø, Nedre Domen, lake and pond at road E75, 70.3215°N; 31.0341°E, 120 m a.s.l., 05.ix.2010, leg. Alyssa Anderson; 1 Norway, Finnmark, Nordkapp, Nordkapp-Plateau, 71.1446°N; 25.7641°E, 220 m a.s.l., 01-ix-2010, leg. Trond Andersen; 1 Norway, Oppland, Dovre, Rondane National Park, Vidjedalsbekken (upper), Malaise trap, 61.9717°N; 9.83606°E, 1280 m a.s.l., 15.ix.2008, leg. Terje Hoffstad.

#### Etymology.

The species is named “autumnalis”, Latin adjective meaning belonging to autumn, referring to the time of the year when the type material was collected.

#### Diagnosis.

Gymnometriocnemus (Raphidocladius) autumnalis can be separated from other *Gymnometriocnemus* species by the following combination of characters in the adult male: body brown, dark brown; virga long and conspicuous with strong lateral sclerotization, anal tergite without dorsal anal point or ridge, gonostylus with convex outer margin and well-defined median crista dorsalis.

#### Description.

Male adult (n = 4 unless otherwise stated). Wing length 1.30–1.52, 1.43 mm. Colouration completely brown, dark brown except for pale transverse bands posteriorly on abdominal tergites V–VIII, narrower on tergite V.

Head. Antennal ratio in Table 1. Temporal setae 8-10, 9; palp lengths (in µm): 25/35/80-90/75-90/105-125.

Thorax. Antepronotum with 2 setae. Dorsocentrals 10-11; acrostichals 8-9, minute and difficult to discern; prealars 4-6; scutellars 2-5.

Wing (Fig. 5D). Costa moderately extended, not reaching half way to apex of M_1+2_; R_2+3_ approaching costa at ½ distance between R_1_ and R_4+5_. Macrotrichia frequent on membrane in apical 1/3 of wing, absent from cell m, few (0-6) in cells cu+an. Veins Sc, R_2+3_, M, Cu, PCu and pseudovein without setae.

Legs. Fore tibia with one spur, 40 µm long; mid tibia with two spurs ca. 20 µm long; hind tibia with well-developed comb and 2 spines, ca. 50 µm long. Fore leg ratios in Table 1.

Hypopygium (Fig. 4D). Ninth tergite with 16-19, 18 setae, without anal point or ridge. Virga 110-120, 115 µm long consisting of two spines, with strong lateral sclerotization. Inferior volsella well-developed lobe; gonostylus with slightly convex outer margin and well-defined median crista dorsalis.

Female and immature stages unknown.

#### Remarks.

The species is morphologically similar to Gymnometriocnemus (Raphidocladius) brumalis and Gymnometriocnemus (Raphidocladius) kamimegavirgus, but different in lacking an anal point or ridge on the anal tergite. This character is similar to characters reported for *Gymnometriocnemus terrestris* and *Gymnometriocnemus tairaprimus*, but these two species can according to original descriptions be separated by having a higher AR (1.4 in *Gymnometriocnemus terrestris*) and a different shape of the superior volsella (Krüger and Thienemann 1941; Sasa and Okasawa 1994). The virga of *Gymnometriocnemus terrestris* has not been described, but the species is listed as a member of subgenus *Gymnometriocnemus* in the World Catalogue of Chironomidae (Ashe and O’Connor 2012). We have examined two males and one female between cellophane strips on two separate pins from the Goetghebuer collection; labels reading “Allemagne, Dr Thienemann, Bonn 1939”. Only one pin with a female bears a type label, but all specimens are likely part of the type series and regarded as syntypes. A small virga is visible in a rather well mounted and cleared hypopygium on one of the specimens. We can confirm placement in subgenus *Gymnometriocnemus* and that none of the specimens we have collected in Norway belong to this species. Gymnometriocnemus (Raphidocladius) autumnalis has so far only been recorded from the very north of mainland Norway and the Rondane mountains (1280 m a.s.l.) in Central Norway.

### Gymnometriocnemus (Raphidocladius) brumalis

Taxon classificationAnimaliaDipteraChironomidae

(Edwards, 1929)

#### Remarks.

We have barcoded specimens from eastern, central and northern Norway that fall within the same genetic cluster although with quite large intraspecific divergence (0–6.8%, mean 3.42% K2P-distance) (Fig. 6). We are at present not able to find morphological differences that fully correspond to the internal groupings inside the Gymnometriocnemus (Raphidocladius) brumalis cluster and all our specimens fit the description by Sæther (1983) with the additions by Cranston and Oliver (1988). There is some variation observed in the length of the virga in relationship with the length of the gonocoxite, one of the characters used to separate Gymnometriocnemus (Raphidocladius) acigus from Gymnometriocnemus (Raphidocladius) brumalis (Cranston and Oliver 1988; Sæther 1983), but we have not examined or barcoded a sufficient number of specimens to confidently conclude if the divergent genetic clusters in our Gymnometriocnemus (Raphidocladius) brumalis can be regarded as separate species. We have examined the male holotype and a male paratype of Gymnometriocnemus (Raphidocladius) acigus and can confirm that the species fits our and Cranston and Oliver’s (1988) interpretation of Gymnometriocnemus (Raphidocladius) brumalis. The species has a Holarctic distribution.

### Gymnometriocnemus (Raphidocladius) kamimegavirgus

Taxon classificationAnimaliaDipteraChironomidae

Sasa & Hirabayashi, 1993

Gymnometriocnemus kamimegavirgus Sasa & Hirabayashi (Sasa and Hirabayashi 1993; Sasa and Okasawa 1994).
Gymnometriocnemus volitans (Goetghebuer), misidentifications (e.g. Brundin 1956).
Gymnometriocnemus (Raphidocladius ?) volitans (Goetghebuer) sensu Brundin (1956), misidentification (Sæther 1983).
Gymnometriocnemus (Raphidocladius) volitans (Goetghebuer) sensu Brundin (1956) (Ashe and O’Connor 2012; Sæther and Spies 2013).

#### Diagnosis.

Gymnometriocnemus (Raphidocladius) kamimegavirgus can be separated from other species of the genus *Gymnometriocnemus* by having well-developed, long virga (about the length of the gonocoxite); AR 0.9-1.1 (n=5); LR_1_ about 0.53-0.56 (n=3); wing membrane with setae at the apex only, occasionally with 1-2 setae proximally in cell an; R_2+3_ situated in the middle between R_1_ and R_4+5_; dark brown almost blackish thorax and head, slightly paler abdomen and legs.

#### Remarks.

Our examined material is from eastern, central and northern Norway, frequently collected near streams, rivers and moors. Male adults fit well with Brundin’s description of *Gymnometriocnemus volitans*, and Sasa & Hirabayashi’s description of *Gymnometriocnemus kamimegavirgus* except for slightly fewer setae on the abdominal tergites (Brundin 1956; Sasa and Hirabayashi 1993; Sasa and Okasawa 1994). The species is Holarctic in distribution.

### Key to Holarctic male adults of the genus *Gymnometriocnemus*

The species *Gymnometriocnemus brevitarsis* is only known as female and therefore not included in the key.

**Table:** 

1	Large, needle-like virga well-developed, sometimes with strong lateral sclerotization (Fig. 4D–F)	**2**
–	Virga small and inconspicuous, without lateral sclerotization (Fig. 4A–C)	**5**
2	Anal tergite without ridge or anal point (Fig. 4D)	**3**
–	Anal tergite with at least a median triangular ridge (Fig. 4E, F)	**4**
3	Inferior volsella with obvious dorsal and ventral lobe (Japan)	**Gymnometriocnemus (Raphidocladius) tairaprimus**
–	Inferior volsella with single lobe (Norway) (Fig. 4D)	**Gymnometriocnemus (Raphidocladius) autumnalis**
4	Wing membrane with setae on wing tip only; often strong sclerotization laterally of virga (Fig. 5F)	**Gymnometriocnemus (Raphidocladius) kamimegavirgus**
–	Wing membrane with setae on at least half of wing; no strong sclerotization laterally of virga (Fig. 5E)	**Gymnometriocnemus (Raphidocladius) brumalis**
6	Body pale yellow-green with pale brown scutal markings	**Gymnometriocnemus (Gymnometriocnemus) pallidus**
–	Body completely brown, or when yellowish ground colour with dark brown scutal markings	**7**
7	Edge of anal tergite broadly rounded (possibly an anal point, but difficult to discern in syntypes); gonostylus strongly curved inwards	**Gymnometriocnemus (Gymnometriocnemus) terrestris**
–	Anal point present, triangular; gonostylus at most with a weakly convex outer margin (Fig. 4A, C)	**8**
8	Ground colour of thorax yellowish (Japan)	**Gymnometriocnemus (Gymnometriocnemus) johanasecundus**
–	Ground colour of thorax brown	**9**
9	Anal point moderately well developed, c. 38 µm long (Fig. 4A)	**Gymnometriocnemus (Gymnometriocnemus) marionensis**
-	Anal point weakly developed, c. 17 µm long (Fig. 4C)	**Gymnometriocnemus (Gymnometriocnemus) subnudus**

## Final remarks

As a result of this study, there are now 17 species of *Gymnometriocnemus* registered worldwide and the genus is present in all major biogeographical regions except Antarctica. Our findings through moderate sampling in Norway indicate that the number of species could be considerably higher also on a global scale and show that molecular data can be a great advantage in diversity assessments of targeted groups. Moreover, our study also highlights the importance of consulting type material for correct identification of Chironomidae if we are to avoid long term misconceptions of species.

## Supplementary Material

XML Treatment for Chaetocladius volitans

XML Treatment for Gymnometriocnemus brevitarsis

XML Treatment for Gymnometriocnemus (Gymnometriocnemus) marionensis

XML Treatment for Gymnometriocnemus (Gymnometriocnemus) pallidus

XML Treatment for Gymnometriocnemus (Gymnometriocnemus) subnudus

XML Treatment for Gymnometriocnemus (Raphidocladius) autumnalis

XML Treatment for Gymnometriocnemus (Raphidocladius) brumalis

XML Treatment for Gymnometriocnemus (Raphidocladius) kamimegavirgus

## Supplementary materials

Supplementary material 1 Examined and DNA barcoded *Gymnometriocnemus* species from Norway Data type: Excel spreadsheet with specimen data and GenBank accessions Explanation note: The spreadsheet contains the following information for all examined species: sample id, catalogue number, voucher status, locality, date of collection, collector and GenBank accession. This dataset is made available under the Open Database License (http://opendatacommons.org/licenses/odbl/1.0/). The Open Database License (ODbL) is a license agreement intended to allow users to freely share, modify, and use this Dataset while maintaining this same freedom for others, provided that the original source and author(s) are credited.Elisabeth Stur, Torbjørn Ekrem
